# Draft Genome and Plasmid Sequences of *Chlamydia pneumoniae* Strain B21 from an Australian Endangered Marsupial, the Western Barred Bandicoot

**DOI:** 10.1128/genomeA.01223-13

**Published:** 2014-02-06

**Authors:** Eileen Roulis, Nathan Bachmann, Adam Polkinghorne, Margaret Hammerschlag, Stephan Kohlhoff, Peter Timms

**Affiliations:** a Institute of Health and Biomedical Innovation, Queensland University of Technology, Kelvin Grove, Queensland, Australia; b Department of Pediatrics, SUNY Downstate Medical Center, Brooklyn, New York, USA

## Abstract

*Chlamydia pneumoniae* is a ubiquitous intracellular pathogen, first associated with human respiratory disease and subsequently detected in a range of mammals, amphibians, and reptiles. Here we report the draft genome sequence for strain B21 of *C. pneumoniae*, isolated from the endangered Australian marsupial the western barred bandicoot.

## GENOME ANNOUNCEMENT

*Chlamydia pneumoniae* is an obligate intracellular bacterium associated with community-acquired pneumonia and detected in cold- and warm-blooded hosts such as amphibians, reptiles, marsupials, and humans (1–3). Although minor genetic differences suggest that human strains are a result of several zoonotic events in this pathogen’s history, both human and koala strains of *C. pneumoniae* are highly conserved in their gene content and organization (4). Interestingly, a previous study examining selected molecular targets in marsupial *C. pneumoniae* isolates demonstrated identical sequences for all isolates tested (5). With this in mind, we sequenced the chromosome and plasmid of B21, an isolate from the western barred bandicoot (*Perameles bougainville*), an endangered Western Australian marsupial.

The *C. pneumoniae* B21 chromosome and plasmid were sequenced in duplicate using the Ion Torrent platform P1 chip with 200-bp chemistry, resulting in over 120 million reads. Read quality analyses were performed using FASTQC version 0.10.1 (Babraham Bioinformatics group [http://www.bioinformatics.bbsrc.ac.uk/projects/fastqc]), with average Phred scores of 24. CLC Genomics Workbench (version 6.0.2; CLC bio, Denmark) was used to trim sequences as well as perform *de novo* assembly and readmapping, with read coverages exceeding 5,000×. The *de novo* assembly consists of 164 chromosomal contig sequences and a single contig for the plasmid. The predicted length of the *C. pneumoniae* B21 chromosome is 1,241,009 bp, with GC content of 40.5%. The B21 plasmid is 7,529 nucleotides (nt) in length, with a GC content of 33.0%. Automatic annotation was performed using RAST (6) and manually curated using Artemis (7).

The draft B21 chromosome and plasmid were pairwise aligned to all available *C. pneumoniae* genome and plasmid sequences (4, 8–11) by use of Multiple Alignment using Fast Fourier Transform (MAFFT) (12). The B21 chromosome contains 1,308 predicted coding sequences (CDS) with 37 tRNAs and a single rRNA operon, while the B21 plasmid has eight predicted CDS. Nucleotide variants were predicted by mapping the reads of *C. pneumoniae* B21 to the complete genome of *C. pneumoniae* LPCoLN, a koala strain. Twenty-six variants were predicted, of which 15 are within homopolymeric tracts of various lengths. Four variants are predicted within intergenic regions. PCR and sequencing analysis of four variants has demonstrated identical sequences to those of LPCoLN. The B21 chromosome is 99.99% identical to that of LPCoLN and 96.9% identical to human strains. Two open reading frames (ORFs) are predicted for the pmp18 (pmpE/F) B21 homolog, which are full length in human strains and a pseudogene in LPCoLN. The B21 plasmid has 99.99% nucleotide identity to the LPCoLN plasmid and 96.3% identity to the horse *C. pneumoniae* N16 plasmid isolate (13). A single nucleotide deletion is noted at the 3′ end of locus X556p_1186 in the B21 plasmid within a homopolymeric tract, resulting in the truncation of X556p_1186 in B21 by 8 amino acids. X556p_1186 is predicted to encode pGP3-D, a putative virulence factor associated with growth of *Chlamydia* in mammalian cells (14).

### Nucleotide sequence accession numbers.

This whole-genome shotgun project has been deposited at DDBJ/EMBL/GenBank under the accession number AZNB00000000. The version described in this paper is version AZNB01000000.
